# Prevalence and associated risk factors of HIV in prisons in Balochistan, Pakistan: a cross-sectional study

**DOI:** 10.12688/f1000research.16994.2

**Published:** 2019-03-15

**Authors:** Muhammad Dawood Khan, Ahmad Wali, Razia Fatima, Aashifa Yaqoob, Shoaib Aziz

**Affiliations:** 1 Department of Health, Provincial AIDS Control Program, Quetta, Balochistan, 87300, Pakistan; 2 Department of Health, Provincial TB Control Program, Quetta, Balochistan, 87300, Pakistan; 3 Ministry of National Health Services, Regulation and Coordination, National TB Control Program, Islamabad, Pakistan

**Keywords:** Prison, HIV, AIDS, prevalence, risk-factors.

## Abstract

**Background: **The prevalence of HIV is 0.1% in Pakistan, with epidemicity in high-risk groups. The infection is on verge of transmission from key populations to the general population through people who inject drugs and sexual transmission. Prisoners are vulnerable to infectious diseases like HIV. This study was conducted in four prisons in Balochistan, Pakistan to determine the prevalence of HIV and associated risk factors.

**Methods: **This cross sectional study was conducted from March to June 2018, in the prisons of Balochistan. WHO-approved rapid diagnostic kits were used for determining the prevalence of HIV and structured interviews were conducted for the assessment of risk factors.

**Results: **Out of 2084 screened prisoners, 33 (1.6%) were found to be positive. A subset of 104 interviews was analyzed for risk factors of HIV. Among HIV-infected prisoners 68.8% (OR 4.48; 95% CI 1.41-14.2) had extramarital sex, 43.8% (OR 2.09 95% CI 0.69-6.28) had a homosexual experience, and 50% had a history of needle sharing (OR 43; 95% CI 7.77-237). About 94% (OR 16.42; 95% CI 2.09-129.81) of prisoners had a history of drug addiction of any type while 50% (OR 13; 95% CI 2.82-60.01) of HIV-infected had a history of using injectable drugs. Around 75% of HIV-infected prisoners had spent 1-5 years in prison, and 25% had spent more than 10 years.

**Conclusion: **The high prevalence of HIV in prisons of Balochistan demands that preventive and treatment strategies should be designed and implemented carefully, allowing early diagnosis and treatment initiation to minimize the spread of infection among the prisons and ultimately their onward transmission into the community.

## Introduction

Globally; about 36.7 million people were living with HIV. HIV infection causes AIDS and is epidemic throughout the world. In 2016; about 1.8 million were reported as newly infected and 1 million died from AIDS related illnesses in the same period ( http://www.unaids.org). In Pakistan, an estimated 0.13 million peoples are infected with HIV, out of which only 17% are registered with the AIDS Control programme and 54% of total registered people currently living with HIV/AIDS are getting treatment from antiretroviral therapy (ART) centres ( http://www.nacp.gov.pk). The prevalence rate of HIV is 0.1% in Pakistan ( http://www.unaids.org). AIDS was limited and epidemic among key populations over the decades in Pakistan, but the latest evidence suggests that there is a shift from key populations through transmission via needle-sharing by injecting drug users (IDUs) to sexual transmission via multiple sexual partners, and further transmission to their intimate partners is increasing ^1^. According to Integrated Biological and Behavioural Survey (IBBS), HIV prevalence was 38.4% among people who inject drugs (PWIDs), about 7.1% among transgender (TG), 3.5% among men who have sex with men and 2.2% among female sex workers ^1– 3^.

Prisons are the easily approachable venues for health interventions but unfortunately, the prisoners are among the most marginalized and restricted populations where access to health services, interventions and surveys are very limited ^4^. Prisoners are one of the most vulnerable population for acquiring infectious diseases especially HIV because people from diverse background, communities and risk factors are kept together for varying time ^5^. The prevalence of HIV is alarmingly increasing in PWIDs and evidence through IBBS indicated that 38.5% of PWIDs were arrested in past 12 months which makes the other prisoners also vulnerable as PWIDs have access to drugs in the prisons ^2^. A study of HIV-positive prisoners in five jails in Sindh, Pakistan indicates that 12.3% of jail prisoners have ever used drugs by injection in the jails ^6^.

The revised Pakistan AIDS Strategy III, developed in 2017 by the National AIDS Control Program (NACP) to guide Pakistan’s overall national response for HIV and AIDS till 2020, through focused interventions with set targets, costs, roles and responsibilities has emphasized on adopting precision targeting (including prisoners) to achieve the required level of impact on the epidemic ^1^. The Balochistan AIDS Control Program (BACP) conducted awareness and screening of prisoners in Central Prison Gaddani in 2017 and found that 27 (6.85%) out of 394 prisoners were positive for HIV. Most of the studies from the country have found that HIV is prevalent at about 2% in prisoners which is much higher than the prevalence in the general population ^4, 5, 7– 13^.

Availability of limited data across the different prisons of the province and high prevalence of HIV found in central jail Gaddani warranted evidence to be produced by conducting a detailed study in other prisons of Balochistan as well. The study aimed to assess the prevalence and risk factors of HIV in prisoners of four major prisons of Balochistan.

## Methods

### Study design and setting

A descriptive cross-sectional study was carried out from March to June 2018 in four major prisons of Balochistan i.e. Quetta, Gaddani, Mach and Loralai. Balochistan is the most widely spread and less populated province of Pakistan with the population of 12.3 million ( http://www.pbs.gov.pk). Area wise it is the largest province and covers 347,190 km ^2^ and comprises of 33 districts ( http://www.balochistan.gov.pk). The province is bordered by Afghanistan in the north and north-west, Iran in the south-west, Khyber Pakhtunkhwa province in the north-east and Punjab and Sindh provinces in the east.

BACP is one of the vertical programs of the health department, providing preventive and curative services to high-risk population in the province. The program has two treatment centres (ART Centres) located in one of the largest tertiary care hospital (Bolan Medical Complex Hospital) at provincial capital Quetta and District Headquarter (DHQ) hospital of Kech, in the city of Turbat. The program is also running 30 screening centres in DHQ Hospitals. Patients with HIV are referred to ART centres for registration and treatment provision from all the screening centres and other health care facilities of the province.

***Specific settings.*** There are 11 prisons in Balochistan of which three are central prisons and the remaining eight are district jails ^14^. Jails are meant to keep the prisoners who are under trial or imprisoned for short durations, whereas prisons are meant to keep the prisoners who are imprisoned for long duration. In Balochistan the jail may serve as prison and prison may serve as jail due to limited capacity for prisoners and long distances from the trial court. The four prisons were selected on the basis of number of prisoners and type of jail, as these prisons cater the majority of the prisoners from Balochistan.

The provincial program started 6-month-long service delivery packages (SDPs) in four selected prisons of Balochistan from March to August 2018. In these SDPs, screening as well as treatment and preventive services were provided to jail prisoners.

### Study population/participants

The study included all the prisoners enrolled at the time of screening irrespective of their status or duration of imprisonment, age, sex, marital status, education status, history of drug addiction, place of origin and employment status. Prisoners were screened for HIV after obtaining written informed consent. Blood specimen collection and screening were conducted in the health facility of jails. Interviews were conducted for assessment of risk factors with HIV. The prisoners found positive on screening were linked with treatment centres in order to commence treatment procedures.

### Data collection methods

Data was collected by a team of trained data collectors that was already involved in screening and provision of SDPs in the mentioned prisons.

Screening was conducted through World Health Organization (WHO)-approved rapid diagnostic kits for HIV Screening (Alere Determine HIV-1/2 Ag/Ab Combo, with sensitivity >99%) provided by the Balochistan AIDS Control Program. The HIV status of HIV-positive prisoners was confirmed by initial confirmatory rapid diagnostic kits (Uni-Gold HIV with Specificity > 99%) and followed by confirmatory rapid diagnostic kits (SD Bioline HIV-1/2 3.0 with Sensitivity > 99%, Specificity > 98%) ^15^.

The risk factors identification for HIV were assessed through structured a questionnaire developed, pretested and validated by the United Nations Office on Drugs and Crime (UNODC), available on OSF ^19^. The interviews primarily were not part of initial agreement between BACP and partner organization delivering the SDPs, but keeping in view the large number of HIV-positive prisoners found in Gaddani prison, they were added to assess the risk factors for HIV. For this purpose, interviews were conducted in specially dedicated visits of the prisons. A total of 150 prisoners were approached to take part in the structured interview, out of which 135 prisoners gave their written informed consent to this aspect of the study. Out of 135 interviews conducted 104 were selected for final analysis while the remaining 31 were discarded due to lack of required information and incomplete filling. A total of 16 out of 104 (15.3%) interviewed prisoners were positive for HIV.

The screening and interview processes were randomly monitored by principal investigator to ensure the quality of data collection. 

### Data variables collected

The outcome variables of the study included the total number of prisoners screened for HIV and the number and proportion of prisoners found positive for HIV. The presumptive exposure variables assessed were age, sex, education and occupation status of prisoners, type of imprisonment and time spent in imprisonment, ever been arrested for drug related offense (Y/N), marital status and extramarital sex status including homosexual sex status (Y/N), sharing needles, razors and blades (Y/N), history of drug addiction (Y/N) and injectable drug addiction (Y/N) and history of blood transfusion, surgery, dental procedure and tattooing (Y/N).

### Statistical analysis

Data was double-entered into Epi-Data software (version 3.1) and analysed in Epi-Analysis (version 2.2.2.183 for analysis, EpiData Association, Odense, Denmark). Descriptive statistics were used to describe the data. Odds ratio (OR) with 95% confidence interval (CI) was calculated for possible presumptive variables.

### Ethical approval

The SDPs had already commenced in the selected prisons before the start of this study by BACP, with the permission of the Health Department of Balochistan and Home Department Government of Balochistan. Screening, health education sessions and collection of data was routinely done for the SDPs so the program data was utilized. The principal investigator was also the monitor of SDPs from BACP. Study and ethical approval was obtained from BACP (No. BACP: 01/2018-432) to collect and analyse the program data for the research purpose.

## Results

### Initial screening and demographic information

**Figure 1.**
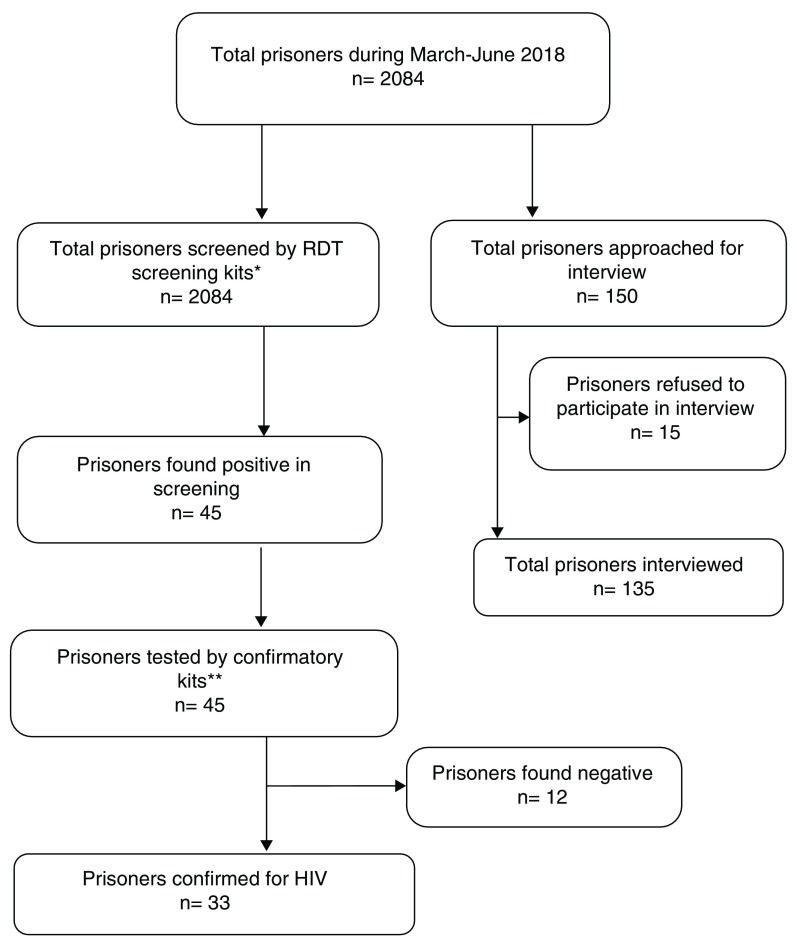
Flow diagram of prisoners screened and interviewed for HIV in prisons in Balochistan. *Test 1 = Alere Determine HIV-1/2 Ag/Ab Combo for detection of Antigent/Antibody, **Test 2 = Uni-Gold HIV, **Test 3 = SD Bioline HIV-1/2 3.0.

A total of 2084 prisoners were screened, of whom 33 were found to be positive for HIV ( Figure 1). All the HIV-positive prisoners were approached for assessment of risk factors but only 16 gave informed written consent for the interview.

The median age of the prisoners was 25 years (range 13–80 years, 94.23% in 15–45 years age group) years and 53 (50.9%) of them were married. The majority of them were uneducated (36.53%) or had been in education for less than 5 years (16.34%). Among them, 15% were unemployed and 8% were working on daily wages before the imprisonment. Around 93% of the prisoners were from Balochistan, 4% from other provinces and 3% from other countries ( Table 1).

**Table 1.** Sociodemographic characteristics of prisoners interviewed for assessment of risk factors for HIV.

Variable	HIV positive, n (%)	HIV negative, n (%)	Total, n (%)
Total	16 (15.4)	88 (84.6)	104
Age, years			
<15	0	1 (0.96)	1 (0.96)
15–45	16 (100)	82 (78.8)	98 (94.2)
45–65	00	02 (1.92)	02 (1.92)
65 and above	00	03 (2.88)	03 (2.88)
Sex			
Male	16 (100)	88 (100)	104 (100)
Place of origin			
Within province	16 (100)	81 (77.8)	97 (93.2)
Out of province	00	04 (3.84)	04 (3.84)
Out of country	00	03 (2.88)	03 (2.88)
Education			
Not educated	09 (56.3)	29 (33.0)	38 (36.5)
<5 years	01 (6.25)	16 (18.1)	17 (16.3)
6–10 years	02 (12.5)	20 (22.7)	22 (21.2)
11–14 years	04 (25.0)	20 (22.7)	24 (23.1)
>14 years	00	02 (2.27)	02 (1.92)
Religious	00	01 (1.13)	01 (0.96)
Occupation			
Unemployed	02 (12.5)	14 (15.9)	16 (15.3)
Employed *	12 (75.0)	68 (77.3)	80 (76.9)
Daily wages	02 (12.5)	06 (6.81)	08 (7.69)
Marital status			
Unmarried	10 (62.5)	41 (46.6)	51 (49.0)
Married	06 (37.5)	47 (53.4)	53 (51.0)

*Employed includes prisoners who have worked both in public, private sector and/or self-employed.

Around 75% HIV infected prisoners were convicted, 75% had spent less than five years, 25% had spent more than ten years in prisons. 22% of total interviewed prisoners had been ever arrested for drug related offense ( Table 2).

**Table 2.** Imprisonment status of prisoners in prisons setting of Balochistan evaluated during March-June 2018 (n=104).

Variable	HIV positive, n (%)	HIV negative, n (%)	Total, n (%)
Total	16 (15.4)	88 (84.6)	104
Type of imprisonment			
Under trial	03 (18.8)	26 (29.5)	29 (27.8)
Convicted	13 (81.3)	62 (70.5)	75 (72.1)
Time imprisoned, years			
< 1	08 (50)	37 (42.0)	45 (43.2)
1–5	04 (25)	34 (38.6)	38 (36.5)
6 – 10	00	12 (13.6)	12 (11.5)
> 10	04 (25)	05 (5.7)	09 (8.65)
Ever arrested for drug- related offense			
No	10 (62.5)	71 (80.7)	81 (77.8)
Yes	06 (37.5)	17 (19.3)	23 (22.1)

### Information surrounding HIV risk factors

Extramarital sex was reported by 11 (68.8%) out of 16 HIV-positive prisoners (OR 4.48; 95% CI 1.41–14.2). Homosexuality was reported by 7 (43.8%) HIV-positive prisoners (OR 2.09; 95% CI 0.69–6.28). About 50% of HIV positive prisoners had history of needle sharing (OR 43; 95% CI 7.77–237.87). Almost 94% of HIV positive prisoners had history of drug addiction of any type, of whom 50% had history of injectable drugs (OR 13; 95% CI 2.82–60.01). History of sharing razorblades and blood transfusion was 9.6% in HIV-positive prisoners; 12.5% had gone through any surgical procedure, 8.6% through any dental procedure and 6.7% had history of tattooing in the past 5 years ( Table 3).

**Table 3.** Risk factors associated with prevalence of HIV in prisons setting of Balochistan. 2018.

Variable	HIV positive, n (%)	HIV negative, n (%)	OR	95% CI
Total	16 (15.4)	88 (84.6)		
Extramarital sex status	11 (68.8)	27 (30.7)	4.48	1.41–14.2
Homosexual sex status	07 (43.8)	22 (25)	2.09	0.69–6.28
Sharing needles	08 (50)	02 (2.3)	43	7.77–237.87
Sharing razors and blades	03 (18.8)	07 (8)	2.67	0.61–11.66
History of drug addiction	15 (93.7)	42 (47.7)	16.42	2.09–129.81
Type of drug addiction				
Injectable drugs	08 (50)	03 (7.1)	13	2.82–60.01
Other drugs	08 (50)	39 (44.3)		
History of blood transfusion	00 (00)	10 (11.3)	00	
History of surgery	02 (12.5)	11 (12.5)	01	0.2–5.01
History of dental procedure	00 (00)	09 (10)	00	
History of tattooing	02 (12.5)	05 (5.68)	2.37	0.42–13.44

OR, odds ratio; CI, confidence interval.

## Discussion

This study was conducted in four major jails in Balochistan, Pakistan, and revealed that the HIV prevalence was 1.6%, which is much higher than the prevalence rate in general population in Pakistan ^3^. A previous study conducted in Quetta jail indicates that the HIV prevalence was 0.5% ^16^. A limitation of that study was generalizability to the whole province as the sample size was very small (only 200 prisoners were screened) and the prisoners of Quetta jail, although large in number, were mostly under trial, so the long-term sentenced prisoners were excluded from being assessed. The study findings are consistent with studies conducted in other provinces where the HIV prevalence ranged from 1 to 2.9% ^4, 5, 7, 9, 11, 13^.

Based on our study findings, the majority of the HIV-positive prisoners belonged to the 15–45 years age group. The same age range was also found to be significant in other studies conducted in Pakistan ^5, 11, 13^. Education status was also found as an important factor in HIV risk assessment, as a large majority of HIV positive prisoners (62.5%) were not educated or had been educated for less than 5 years, as found by another study conducted in Lahore ^11^.

The prisoners who were single had a higher prevalence of HIV, as 62.5% of HIV-positive prisoners were unmarried. The findings are consistent with a study conducted in different jails of six cities in Sindh province ^13^, Imprisonment status and duration of imprisonment was significant as 81% of HIV-positive prisoners were convicted and 25% prisoners had spent more than 10 years in prisons.

A substantial proportion of HIV-infected prisoners were involved in behaviour considered to be presumptive risk factors associated with HIV; 68.8% were involved in extramarital sex and 43.8% had engaged in homosexual practices. A study of prisoners in Karachi found involvement of prisoners in homosexuality and sexual encounters with females to be 21.3% and 45.9%, respectively ^4^. Moreover, drug addiction, especially injectable drugs involving the sharing of needles, was also found to be present in significant majority of the HIV infected prisoners. Risk of acquiring of HIV increases in prisons as studies shows that 38.5% of PWID were arrested in past 12 months and around 12.3% of arrested PWID had access to injectable drugs in prisons ^2, 17^.

The young age of the prisoners, being less educated, having low socioeconomic status, addiction to injectable drugs, having multiple sex partners, sharing needles and involvement in drug related offense were notable findings of the study and consistent with studies conducted elsewhere in Pakistan ^4, 5, 7– 13^. 

Prison settings highly favour the spread of infectious disease as the prisoners from different sociodemographic backgrounds like low literacy levels and having pre-imprisonment risk factors such as drug use, extramarital sex encounters etc. are forced to live together in confined space without having recreational facilities ^4^. The prisoners are vulnerable to acquiring new infections in prisons due to overcrowding, poor medical facilities (especially diagnostic investigations) and the unavailability of harm reduction approaches such as the use of condoms or needle exchange program ^18^. All these factors may not only lead to greater chances of spread of infections like HIV among the jails but can also be a major concern among the general population due to frequent flow of prisoners into the community ^4^.

Aside from the many risk factors responsible for spread of infections in prison settings, prisons are still favourable venues for the screening and treatment of HIV, as the prisoners are confined to limited vicinity and easily approachable for diagnosis, treatment and health education. If the prisoners are approached within the prisons for diagnosis and treatment, this can help halt the spread of HIV to the general population and vice versa ^4^.

The level of health services provided in prisons plays an important role in dealing with the associations of HIV-infected persons with the community. In a resource-constrained country like Pakistan, the chain of transmission can be broken by introducing urgent prevention efforts, including health education, initial screening at the time of entry of prisoners in prions, routine periodic screening of all the prisoners and treatment of infected prisoners in each prison so that its spread within the prisons and to the community after release of the prisoner could be minimized ^4^. The presence of HIV-infected prisoners warrants that information and education programs are made accessible in the prisons to stop the spread of infectious diseases in the prisons, with particular emphasis on transmission through injectable drug use and high-risk sexual activities. Training the health care providers to identify and treat those suspected of being, or known to be infected with HIV and the provision of testing services may help in early diagnosis of infections and their treatment to reduce the spread of infection.

Being a comprehensive study covering four major jails of Balochistan province the study is strengthened by universal sampling for screening in which all the prisoners present during the study period were screened for HIV. The risk factors may vary in those prisoners who were not interviewed.

## Conclusion

The high prevalence of HIV in prisons in Balochistan, compared with the non-prison population, indicates that preventive strategies should be designed and implemented carefully to minimize the spread of infection among the prisons and ultimately their linkages to the community. Early diagnosis of HIV infection through screening, initiation of treatment, addressing the stigma universally attached with the disease, peer based, risk reduction education program and linking HIV-positive inmates to the community care after release are effective ways of preventing the spread of infection.

## Data availability

### Underlying data

Raw data for the present study, including demographic information and answers to the questionnaire, is available on OSF, DOI: https://doi.org/10.17605/OSF.IO/8Z4EJ ^19^.

### Extended data

The questionnaire used in this study is available on OSF, DOI https://doi.org/10.17605/OSF.IO/8Z4EJ ^19^.
