# Supplementary material for: Prevalence and associated risk factors of HIV in prisons in Balochistan, Pakistan: a cross-sectional study
Source: F1000Res. 2019 Mar 15;7:1821. Originally published 2018 Nov 20. [Version 2] doi: 10.12688/f1000research.16994.2 (PMC6381798; doi:10.12688/f1000research.16994.2)
Supplement: Peer review history

# Peer review: Reviewer response for version 2

**DOI:** 10.5256/f1000research.20272.r45799

**Published:** 2019-04-23

**Authors:** Pamela Valera

**Affiliations:** 1 Rutgers University School of Public Health, New Brunswick, NJ, USA

Revise the abstract - Introduction -  the prevalence of HIV is very small, but how this is written it sounds more fear mongering given the results that only 1.6% out of 2,084 were HIV+. I would focus on the facts only. The conclusion states - high prevalence compared to whom, please revise? 

 Replace "homosexual experience" with "same-sex experience" in the abstract and entire manuscript. 

 Grammatical errors have not been addressed throughout the paper. For example: " *Prisons are the easily approachable venues for health interventions but unfortunately, the prisoners are among the most marginalized and restricted populations where access to health services, interventions and surveys are very limited".*  This should say - "Prisons are an important venue for health interventions as inmates are among the most marginalized and restricted populations where access to health services, interventions, and survey research are very limited *". *I am unsure that prisons are easily approachable venues for health interventions.

 Describe IRB procedures.

Is the work clearly and accurately presented and does it cite the current literature?

Yes

If applicable, is the statistical analysis and its interpretation appropriate?

Yes

Are all the source data underlying the results available to ensure full reproducibility?

Yes

Is the study design appropriate and is the work technically sound?

Yes

Are the conclusions drawn adequately supported by the results?

Yes

Are sufficient details of methods and analysis provided to allow replication by others?

Yes

Reviewer Expertise:

Prison Health, HIV/AIDS, and Prison Education.

I confirm that I have read this submission and believe that I have an appropriate level of expertise to confirm that it is of an acceptable scientific standard, however I have significant reservations, as outlined above.

# Peer review: Reviewer response for version 1

**DOI:** 10.5256/f1000research.18580.r42871

**Published:** 2019-02-18

**Authors:** Laurent Getaz

**Affiliations:** 1 Division of Prison Health, Geneva University Hospitals & University of Geneva, Geneva, Switzerland; 2 Division of Tropical and Humanitarian Medicine, Geneva University Hospitals & University of Geneva, Geneva, Switzerland

This article describes the prevalence of and risk factors associated with HIV in four prisons in Pakistan. These data are very important for prioritizing programs that are essential for HIV control in this vulnerable population.

 I propose a major revision of this manuscript.

 Methods section:

- How was the HIV screening test proposed in the study population? Was the test optional or mandatory? If the test was not mandatory, what was the participation rate?
- How did you calculate the sample size, in particular for the number of participants who responded to the questionnaire?
- Please clarify the HIV diagnostic algorithm. For participants with a positive "Alere Determine HIV test" result, the HIV status "was confirmed by initial confirmatory rapid diagnostic kits (Uni-Gold) and followed by confirmatory rapid diagnostic kits (SD Bioline)". In the case of a positive "Alere determine test" result and negative "Uni-Gold test" result, did you process the SD Bioline test? How did you manage discordant results?

Discussion section:

- "Education status was also found as an important factor in HIV risk assessment, as a large majority of HIV positive prisoners (62.5%) were not educated or had been educated for less than 5 years". This statement is not true. In the results section, you should provide the results of a bivariate analysis to estimate the association between education level and HIV prevalence. The level of education is not associated with HIV (62.5% versus 51.1%, p=0.4, OR=1.6 (0.5-5.1)). 
- "The prisoners who were single had a higher prevalence of HIV, as 62.5% of HIV-positive prisoners were unmarried". In the results section, you must also provide the results of a bivariate analysis to estimate the association between this variable and HIV. Marital status is not associated with infection (62.5% versus 46.6%, p=0.24). 
- I believe an epidemiologist should be involved as a coauthor to validate the interpretation of the results. You should discuss the result in which a statistically significant association is identified in the study population and put it in perspective with the data from the literature. And when a generally recognized risk factor is not associated with infection in the study population, you should still discuss it. For example, in your study, "extramarital sex status" (OR-4.48, IC95% 1-4-14.2) is associated with HIV but not "homosexual sex status" (OR=2.1, IC95% 0.7-6.3). Also, you cannot state "A substantial proportion of HIV-infected prisoners were involved in behaviour considered to be presumptive risk factors associated with HIV; 68.8% were involved in extramarital sex and 43.8% had engaged in homosexual practices". 
- I suggest you describe the limitations of the study. What potential biases do you suspect, and what are their expected impacts on the results?

The manuscript should be proofread by a native English speaker for spelling, grammar and punctuation, as it contains many errors throughout. Many phrases contain errors, which makes reading the manuscript difficult.

Is the work clearly and accurately presented and does it cite the current literature?

Yes

If applicable, is the statistical analysis and its interpretation appropriate?

Partly

Are all the source data underlying the results available to ensure full reproducibility?

Yes

Is the study design appropriate and is the work technically sound?

Partly

Are the conclusions drawn adequately supported by the results?

Partly

Are sufficient details of methods and analysis provided to allow replication by others?

Partly

Reviewer Expertise:

Tropical medicine / Internal medicine / Public health / Health in detention

I confirm that I have read this submission and believe that I have an appropriate level of expertise to confirm that it is of an acceptable scientific standard, however I have significant reservations, as outlined above.

# Peer review: Reviewer response for version 1

**DOI:** 10.5256/f1000research.18580.r42869

**Published:** 2019-02-11

**Authors:** Pamela Valera

**Affiliations:** 1 Rutgers University School of Public Health, New Brunswick, NJ, USA

There is an urgent public health need to understand HIV/AIDS disease transmission globally especially in areas where war and conflict continues and resources to address communicable diseases are limited.

 This paper is the first to my knowledge to shed light on a growing HIV epidemic especially in correctional facilities in different prisons and jails in Pakistan. The study design provides opportunities for future research to understand HIV/AIDS transmission in correctional facilities. However, I would have much appreciated more literature on the characteristics of offenders prior to incarceration.

 The findings while not generalizable were instrumental in helping readers understand high-risk behaviors among people who inject drugs and people who engage in same-sex behaviors. Perhaps, the next step of this study might be to conduct a qualitative study exploring the lived experience of inmates living with HIV/AIDS in remote correctional settings in Pakistan

 The conclusion points researchers in the right direction. We have a limited understanding of HIV prevalence and HIV risk-taking behaviors of offenders in Pakistan and other areas. We need to do more in understanding HIV prevalence in correctional settings, but also discuss opportunities for prevention intervention programs. Perhaps, a section on implications or future research in the conclusion section should be included.

 Here are potential articles to consider for this section:  

- Scott, D. P., Harzke, A. J., Mizwa, M. B., Pugh, M., & Ross, M. - Evaluation of an HIV peer education program in Texas prisons ^1^. 
- Solomon, L., Montague, B. T., Beckwith, C. G., Baillargeon, J., Costa, M., Dumont, D.,Rich, J. D. - Survey finds that many prisons and jails have room to improve HIV testing and coordination of postrelease treatment ^2^.
- Valera, P., Chang, Y., & Lian, Z. - HIV risk inside US prisons: A systematic review of risk reduction interventions conducted in US prisons ^3^.

Lastly, the manuscript would greatly benefit from proof-reading and addressing grammatical errors throughout the article.

Is the work clearly and accurately presented and does it cite the current literature?

Yes

If applicable, is the statistical analysis and its interpretation appropriate?

Yes

Are all the source data underlying the results available to ensure full reproducibility?

Yes

Is the study design appropriate and is the work technically sound?

Yes

Are the conclusions drawn adequately supported by the results?

Yes

Are sufficient details of methods and analysis provided to allow replication by others?

Yes

Reviewer Expertise:

Prison Health, HIV/AIDS, and Prison Education

I confirm that I have read this submission and believe that I have an appropriate level of expertise to confirm that it is of an acceptable scientific standard, however I have significant reservations, as outlined above.

## References

[1] : Evaluation of an HIV Peer Education Program in Texas Prisons. *Journal of Correctional Health Care*.2004;10(2) : 10.1177/107834580301000203 151-173 10.1177/107834580301000203

[2] : Survey finds that many prisons and jails have room to improve HIV testing and coordination of postrelease treatment. *Health Aff (Millwood)*.2014;33(3) : 10.1377/hlthaff.2013.1115 434-42 10.1377/hlthaff.2013.1115 24590942PMC4028701

[3] : HIV risk inside U.S. prisons: a systematic review of risk reduction interventions conducted in U.S. prisons. *AIDS Care*.29(8) : 10.1080/09540121.2016.1271102 943-952 10.1080/09540121.2016.1271102 28027663PMC5587216

# Response

**Published:** 2019-03-05

**Authors:** Muhammad Khan

**Affiliations:** Provincial AIDS Control Program Balochistan, Pakistan

Dear Pamela Valera

Thank you very much for your valuable feedback. I have tried to remove the grammatical mistakes from the manuscript and amend the conclusion section based on the references provided by you.

# Peer review: Reviewer response for version 1

**DOI:** 10.5256/f1000research.18580.r40898

**Published:** 2018-12-27

**Authors:** Quaid Saeed

**Affiliations:** 1 National AIDS Control Programme, Islamabad, Pakistan

The work quite clearly cites the current and latest literature regarding the HIV disease transmission dynamics in Pakistan and also cites other such studies conducting in different prisons of Pakistan. The study design is appropriate and the study has been conducted in the most difficult and remote prison in Pakistan giving good insight on conditions prevalent in such remote prison settings.

 The study design was appropriate and a good number of prison inmates (2084) were selected. As with other similar studies the prevalence found was more than the one in the general population which warrants that urgent preventive measures should be taken to control spread of this infection in this closed population. In Pakistan, outbreaks of HIV have been reported in different jails of the country in the past. Sensational reports came in the media regarding outbreak of HIV in different prisons but no scientific studies were carried out to determine the reasons of this spread. This study is a good effort to highlight the prevalent conditions and risks for spread of HIV in prison settings.

 The study outlines the risk factors associated with HIV transmission in this population as part of the general population but does not shed ample light on prevailing high-risk behaviours inside the prisons that may have led to transmission of this disease in the prisons. Thus, most of the readers would be interested whether high risk behaviours are prevalent in these prisons and whether drugs and injectable equipment is available in the prisons. Having said that it is not easy to divulge upon these areas since most prison authorities deny prevalence of such practices in their jurisdiction and do not allow such questions to be asked and explored by investigators. It is therefore conceivable that the of this study questionnaire must have been cleared and vetted by the jail authorities and therefore such information could not be explored during this study.

 As far as the methods of analysis was concerned the details provided were quite useful for other investigators to replicate such a study. Details were provided on statistical tools applied and used.

 The conclusion drawn are in line with the findings of the study. We lack data on prevalent behaviours of prison inmates in different prisons in Pakistan and such similar studies are needed to advocate and start prevention interventions in these settings in the country.

Is the work clearly and accurately presented and does it cite the current literature?

Yes

If applicable, is the statistical analysis and its interpretation appropriate?

Yes

Are all the source data underlying the results available to ensure full reproducibility?

Yes

Is the study design appropriate and is the work technically sound?

Yes

Are the conclusions drawn adequately supported by the results?

Yes

Are sufficient details of methods and analysis provided to allow replication by others?

Yes

Reviewer Expertise:

NA

I confirm that I have read this submission and believe that I have an appropriate level of expertise to confirm that it is of an acceptable scientific standard.
